# Evaluation of two independent dose prediction methods to personalize the automated radiotherapy planning process for prostate cancer

**DOI:** 10.1016/j.phro.2022.01.006

**Published:** 2022-02-03

**Authors:** Martijn Kusters, Kentaro Miki, Liza Bouwmans, Karl Bzdusek, Peter van Kollenburg, Robert Jan Smeenk, René Monshouwer, Yasushi Nagata

**Affiliations:** a Department of Radiation Oncology, Radboud University Medical Center, Nijmegen, The Netherlands; b Department of Radiation Oncology, Hiroshima University Hospital, Hiroshima, Japan; c Philips Healthcare, Radiation Oncology Solutions, Fitchburg, WI, USA

**Keywords:** Radiotherapy, Ideal dose distribution, Automated treatment planning, Optimization, Volumetric-modulated arc therapy

## Abstract

**Background and purpose:**

Currently, automatic approaches for radiotherapy planning are widely used, however creation of high quality treatment plans is still challenging. In this study, two independent dose prediction methods were used to personalize the initial settings for the automated planning template for optimizing prostate cancer treatment plans. This study evaluated the dose metrics of these plans comparing both methods with the current clinical automated prostate cancer treatment plans.

**Material and methods:**

Datasets of 20 high-risk prostate cancer treatment plans were taken from our clinical database. The prescription dose for these plans was 70 Gy given in fractions of 2.5 Gy. Plans were replanned using the current clinical automated treatment and compared with two personalized automated planning methods. The feasibility dose volume histogram (FDVH) and modified filter back projection (mFBP) methods were used to calculate independent dose predictions. Parameters for the initial objective values of the planning template were extracted from these predictions and used to personalize the optimization of the automated planning process.

**Results:**

The current automated replanned clinical plans and the automated plans optimized with the personalized template methods fulfilled the clinical dose criteria. For both methods a reduction in the average mean dose of the rectal wall was found, from 22.5 to 20.1 Gy for the FDVH and from 22.5 to 19.6 Gy for the mFBP method.

**Conclusions:**

With both dose-prediction methods the initial settings of the template could be personalized. Hereby, the average dose to the rectal wall was reduced compared to the standard template method.

## 1 Introduction

Generating clinical acceptable plans with treatment planning in the field of radiation oncology is a challenge, because the optimization process is complex and many parameters need to be taken into account [1]. Different approaches have been developed to automate the planning process. Automation of this process helps to improve the consistency of the plan quality and reduce the planning time [2].

Several studies using knowledge-based planning [3], [4], template-based [5], [6] or deep learning-based dose predictions [7], [8] or multicriteria optimization [9] have investigated how different methods can help to further optimize the planning process. Most of these studies regarding prostate cancer cases have shown that automated planning further reduced dose to organs at risk (OARs) compared to manual planning. A review of these published studies can be found in Heijmen et al. [10].

This study focused on optimizing the automated template-based strategy. A template-based strategy starts with a single treatment technique template that is used to define the planning goals for automated treatment plan generation. The most optimal single treatment technique template is usually found by trial-and-error, whereby different possible settings are explored to find a planning template setting that works for all patients. However, since the optimization process run by automated planning algorithms is finite, the initial seed value from which to start the optimization influences the final optimization result. Therefore, this single treatment technique template method does not always automatically lead to the most optimal plan solution and additional manual optimization steps are needed [11]. In Janssen et al. [11] it was shown that an independent knowledge-based QA model can detect clinical plans which can be improved by adapting the settings for the OARs objective goals in the treatment planning template. The goal of this study was to personalize the treatment planning template for prostate cancer treatments by using two different methods to predict the expected dose distribution before planning, to use these predictions to set personalized objectives in a thus personalized treatment planning template and to compare these plans with the current clinical automated single-template based method.

## 2 Materials and methods

### 2.1 Preparation of patient dataset

Datasets of 20 randomly selected clinical high risk prostate patients treated in our clinic were used in this study. All patients had a CT-simulation (3 mm slice thickness) in supine position with knee support and an endorectal balloon within the rectum. All plans were designed to deliver 70 Gy in fractions of 2.5 Gy to the planning target volume (PTV). The clinical target volume (CTV) included the entire prostate with/without the proximal seminal vesicles. The PTV was defined by adding a margin of 5 mm in the posterior direction and 7 mm in all other directions Two 10 MV volumetric-modulated arc therapy (VMAT) arcs were used (95 to 265°) clockwise and counter-clockwise. This beam orientation was set to minimize direct beams go through the rectal and anal wall. All plans were generated for an Elekta Agility^TM^ linac (Elekta Ltd, Crawley, UK). The replanned clinical plans were automatically generated with a single treatment technique template in the Pinnacle^3^ Auto-Planning module in Pinnacle 16.0.2 (Philips Healthcare, Fitchburg, WI, USA). These plans all fulfilled the clinical dose criteria (shown in Table S1 of the supplementary materials) and were used to compare the single template technique with the personalized template technique. The personalized templates were generated by using PlanIQ^TM^ Feasibility DVH (Sun Nuclear Corporation, Melbourne, FL, USA) [12], [13], [14] or the modified filtered back projection method (mFBP) [15], [16]. Both methods predicted personalized dose volume histograms (DVHs) for the OARs before the start of the planning process. By using these predictions the initial optimization parameters were set in a personalized manner and in this way a custom-made automated treatment planning template for each patient was obtained. The clinical plan and the personalized plans were compared by using the dose metrics of V30Gy, V60Gy and mean dose for rectum, anal and rectal wall and V60Gy and mean dose of the bladder and conformity index (CI) and homogeneity index (HI) of the PTV (see supplementary materials for the definition of CI and HI [17]). The Institutional Review Board of our center reviewed and approved this study.

### 2.2 Feasibility DVH method

In Pinnacle 16.2, PlanIQ^TM^ Feasibility DVH was used to estimate the lowest possible DVH for the OARs of any patient, given the full coverage of the target volumes by the prescribed doses and an ideal dose fall-off around the target boundary. More detail about the PlanIQ software can be found in Ahmed et al [12]. The feasibility DVHs were used for setting the objectives for the OARs. The feasibility level (F) can vary between 0 and 1 and needs to be set to determine the DVH and related goals for each OAR. We chose to set the DVH parameters for the anal wall based on feasibility-value (F-value) of 0 (which is on the edge of impossible and most difficult DVH values) and for the bladder and rectal wall an F-value of 0.1 was used (which is on the edge of difficult and DVH values). These values were set to mimic the clinal plan results.

### 2.3 mFBP method

**Fig. 1:**
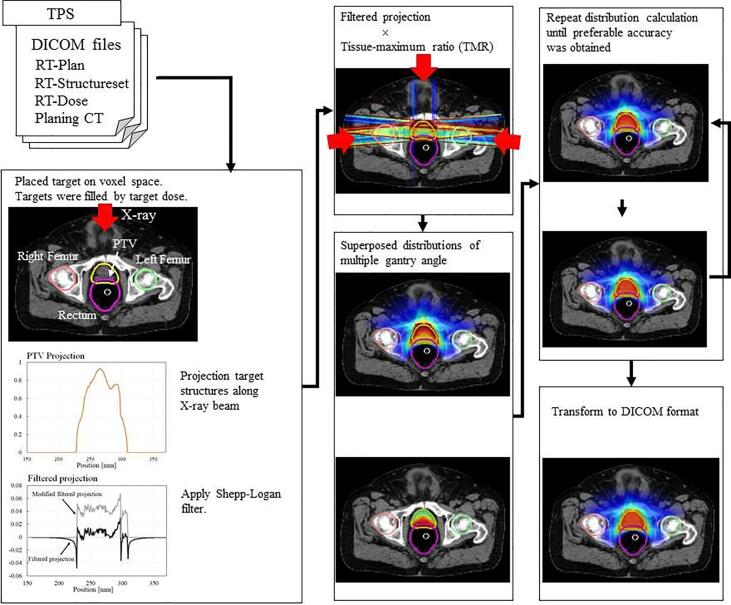
Calculation workflow of mFBP method for prostate cases.

The modified filtered back projection (mFBP) method [15], [16] was used to calculate a geometrically ideal dose distribution that took the geometric beam setup and delineated contours of the PTV into account, as well as body and OARs. A schematic drawing of the mFBP is shown in Fig. 1 and more details can be found in Miki et al. [15]. The predicted dose distribution was translated into DVHs for the OARs. These predicted DVHs were used to individualize the objectives values for the current template technique to acquire the automated dose optimization in a personalized manner for VMAT prostate planning. In the mFBP method a balance between OAR sparing and target coverage had to be set to simulate a clinical feasible dose distribution, therefore the weight settings for the OAR sparing had to be defined. For the OARs the weights for the bladder, rectal wall and anal wall were chosen in such a way that the dose mimics the current clinical plans. The weight were determined on a separate set of 5 clinical prostate cancer plans. As a result of several determinations, weight values w of 0.99, 0.70 and 0.65 were used for the anal wall, rectal wall and bladder, respectively for all cases. Higher values would work as a tighter sparing function. Finally, the calculated dose distribution was normalized in such a way that 99% of the PTV volume is covered by 95% of the dose prescription. The calculated distribution was converted to DICOM dose format. The DICOM dose was imported in our planning system and was used to determine the planning settings of the predicted DVHs for the OARs.

### 2.4 The planning optimization process

The standard Auto-Planning template (see Table 1) was personalized with the predicted DVH parameters. After the Auto-Planning process was finished an extra warm start was performed to steer the PTV coverage in such a way that 99% of the PTV volume is covered by 95% of the total dose of 70 Gy dose prescription.

**Table 1:** Automated planning template is personalized by setting the objective values based on the predicted DVHs for both methods separately in our treatment planning system. R1 and R2 are guidance contours to steer the dose in the surrounding area of PTV + 4 mm at 1 cm and 2 cm distance, respectively.

ROI	Type	Dose (Gy)	Volume (%)	Priority
PTV	Target	70		
Rectal wall - (PTV + 4 mm)	Max DVH	20	10	Medium
Rectal wall - (PTV + 4 mm)	Max DVH	30	7.5	Medium
Rectal wall - (PTV + 4 mm)	Mean Dose	10		Medium
Bladder	Max DVH	60	20	Low
Bladder	Mean Dose	25		Low
Anal wall – (PTV + 4 mm)	Max DVH	5	31	Low
Anal wall – (PTV + 4 mm)	Max DVH	10	10	Low
Anal wall – (PTV + 4 mm)	Mean Dose	6		Low
R1	Mean Dose	35		Low
R2	Mean Dose	20		Low

Advanced settings	
Tuning Balance	0%
Dose Fall-off Margin	2.6cm
Hot-Spot Maximum Goal	104%
Use Cold-Spot ROIs	no

The details for the dosimetric plan comparison can be found in the supplementary materials including the normal tissue complication probability (NTCP) calculation for the risk of rectal bleeding [18]. A statistical analysis was performed by comparing the results of the mFBP with the clinical method and of the FDVH with the clinical method, separately. Normality of the data was tested prior to the analysis. For normal distributed data the paired *t*-test was performed and if not the Wilcoxon paired signed-rank test was chosen. The compared results were considered statistically significant if p<0.05.

The RATING guidelines for treatment planning were used to revise the manuscript. The RATING score for this study was 80% [19].

## 3 Results

**Fig. 2:**
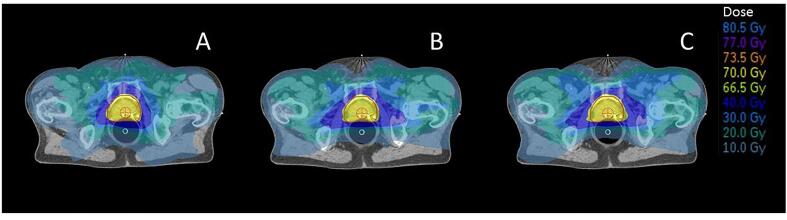
Axial dose distribution of the original Auto-Planning method (panel A) and the two personalized automated planning methods (FDVH, panel B and mFBP, panel C). The colour scale representing different dose levels are shown at the right side of the figure.

The axial dose distributions for the original Auto-Planning method (A) and the two personalized automated planning method FDVH (B) and mFBP (C) are shown in Fig. 2. Dose deposition around the bladder and rectal wall was reduced for both personalized methods, which lead to a different shape of the dose distributions and more spreading of dose in the pelvic bone regions.

Normality test of the dose metrics data was done. No normality for HI and CI was found and for the other dose metrics normality of the distribution were confirmed. As shown in Table 2, there was a small statistically significant difference in median HI for the PTV from 0.07 [0.05–0.09] to 0.06 for MFBP [0.05–0.08] and FDVH [0.04–0.07] method. The median CI of these plans was slightly worse and increased from 1.14 [1.10–1.25] to 1.23 (1.14–1.30) for mFBP and to 1.19 [1.13–1.29] for FDVH, respectively.

**Table 2:** Plan comparison between clinical plans and plans based on the FDVH and mFBP methods. The median dose metrics with range for PTV HI and CI, and average mean dose, percentages for 30 Gy and 60 Gy volumes for rectal wall, anal wall and bladder and MUs are shown for clinical, mFBP and FDVH method. The data marked with an asterisk have p-values lower than 0.05 indicating statistical significance between each personalized method versus the clinical method.

Average/Median dose metrics values											
	PTV HI*	PTV CI*	Rectal wall V30Gy(%)*	V60Gy(%)*	mean dose (Gy) *	Anal wall V30Gy(%)	V60Gy(%)*	mean dose (Gy)	Bladder V60Gy(%)*	mean dose (Gy) *	MUs*
clinical	0.07 [0.05–0.09]	1.14 [1.10–1.25]	27.4	17.0	22.5	6.9	1.9	9.2	10.5	19.8	710
mFBP	0.06 [0.05–0.08]	1.23 [1.14–1.30]	24.2	15.9	19.6	6.9	2.1	9.0	11.6	20.5	771
FDVH	0.06 [0.04–0.07]	1.19 [1.13–1.29]	24.7	16.2	20.1	7.0	2.1	9.1	11.1	20.4	769

For the mFBP method, the average mean dose to the rectal wall was reduced by 2.9 Gy. The average V30Gy and V60Gy for the rectal wall was reduced by 3.2% and 1.1%, respectively. The average V60Gy for the anal wall was increased by 0.2%. For the bladder the average V60Gy and mean dose were increased by 1.1% and 0.7 Gy, respectively.

For the FDVH method the average mean dose to the rectal wall was reduced by 2.4 Gy. The average V30Gy and V60Gy for the rectal wall reduced by 2.6% and 0.7%, respectively. The average V60Gy for the anal wall was increased by 0.2%. For the bladder the average V60Gy and mean dose were increased by 0.7% and 0.6 Gy, respectively.

**Fig. 3:**
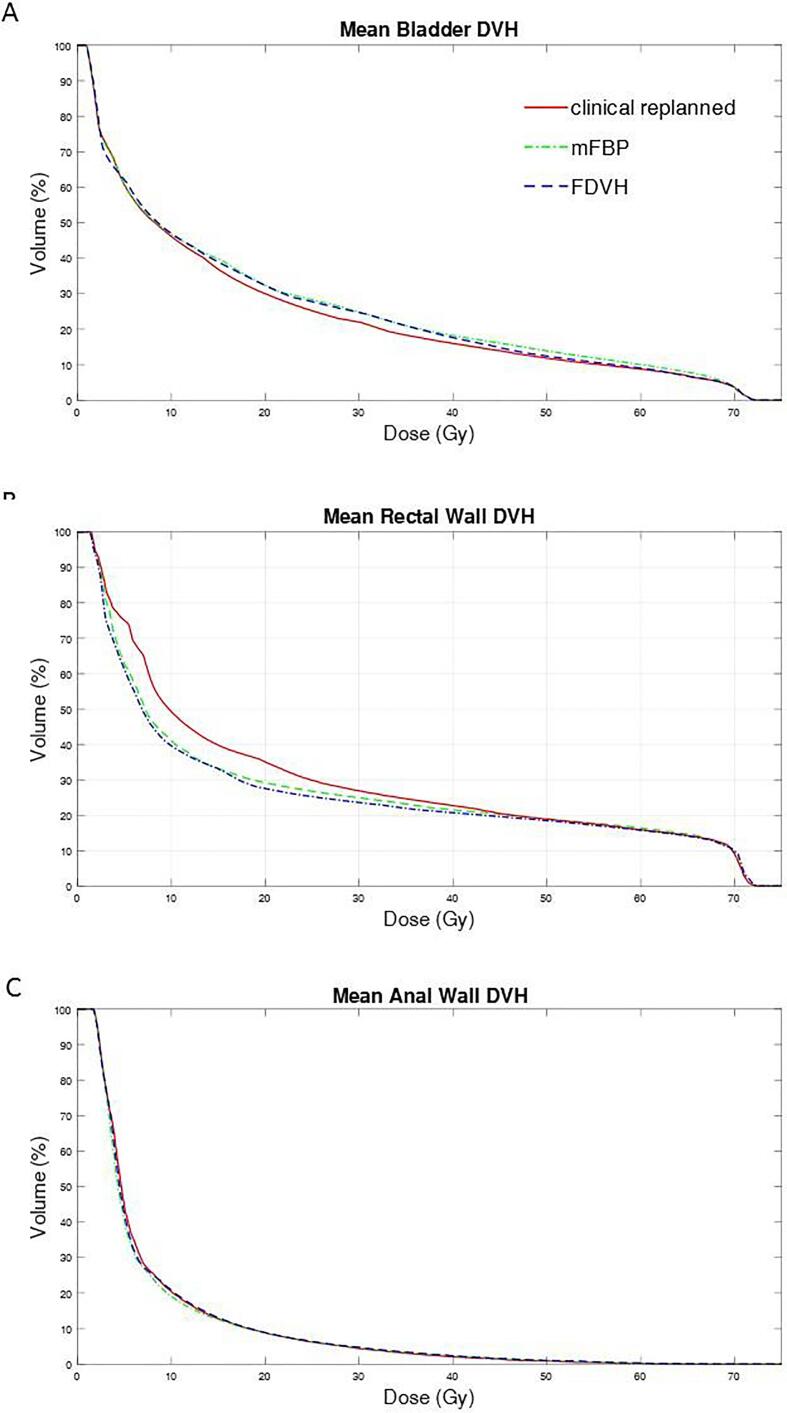
Population mean DVHs for A) bladder, B) rectal wall and C) anal wall are shown for the replanned clinical (solid lines), mFBP (dashed-dotted) and FDVH (dashed lines) plans.

The population mean DVH of the bladder (panel A) was lower for the clinical plans and higher for the rectal wall (panel B) as shown in Fig. 3. No clear difference between planning methods was seen for the population mean DVH of the anal wall (panel C).

The FDVH (769 MUs, range 685–879) and mFBP (771 MUs, range 705–879) methods both had significantly more MUs than the average clinical plans (710 MUs, range 628–795). The increase in MUs is due to an increase of plan complexity and fluence modulation of these personalized plans.

## 4 Discussion

This study compared the current clinical automated single template-based approach with the personalized optimization by setting a personalized template using the predicted DVHs from mFBP and FDVH for automated VMAT planning of prostate cancer treatments. By using an automated personalized template-based treatment strategy it was possible to reduce the dose to the rectal wall.

Various studies presented in a review by Heijmen et al. [10] showed that automated planning for prostate cancer treatment reduced the dose to OARs compared to manual planning. Janssen et al. [11] showed that adapting the single template for each individual patient could further improve the automated plans. This idea was investigated in this study by using the FDVH and mFBP methods to personalize the automated template-based planning approach.

The FDVH method was recently evaluated by Cilla et al. [5] with personalized planning in Pinnacle Evolution 16.4.2 [20] for prostate cancer planning. They also found a significant reduction in mean rectum dose compared with the Auto-Planning plans. In this study the Pinnacle Evolution approach was not investigated, because the comparison could be influenced by the new algorithm as well. In the future, the use of this new algorithm needs to be further explored.

A reduction in rectal wall dose will reduce the risk of late toxicities, like for example rectal bleeding. NTCP for late rectal bleeding is correlated with the volume of the rectal wall that receives a dose of about 64 Gy or higher as shown in Schaake et al. [18]; note that we converted the V70 value with EQD2 formula for rectal wall for a fraction scheme of 2.5 Gy. Since this dose was reduced with both personalized methods, a lower NTCP for late rectal bleeding is expected using the perzonalized templates for automated planning. Using the NTCP formula for the risk of rectal bleeding grade 2 or higher, a reduction of the rectal wall volume that receives a dose of 64 Gy with 1%, the NTCP can be reduced from about 5% to 4% for patients using anticoagulants. When no anticoagulans are used, the NTCP can be reduced from about 1.5 to 1.2%.

It was expected that the personalized methods would reduce the dose for all OARs. However, the results in this study showed only a reduction for the dose to the rectal wall. One of the reasons could be that the clinical template and beam setup were very robust developed and large improvements may not be possible anymore. For the personalized methods a small increase in bladder dose was seen. The reason for the small increase in bladder dose may be due to the change in priority for the rectal wall objectives from low to medium for all plans compared to the current clinical plan in the Auto-Planning template.

Both dose prediction methods are independent of the treatment planning system and can be used for different treatment sites. The results of both methods were comparable. It was expected that the mFBP method would work better than the FDVH method, because the mFBP takes into account the beam setup and the weighting of OARs. However, this was not the case, which may be due to the fact that this method gives a single 3D dose solution which is not yet translated into clinical feasible segments. The FDVH method is also not yet translated into feasible segments, but this method gives a range of solutions for the user to choose from. The settings for the feasibility-values are determined by looking at the DVHs of the OARs of the current clinical plans.

The mFBP method is not integrated in the planning system, which makes it more difficult to implement in the clinic. On the other hand, the FDVH method is available in the treatment planning system, and by choosing the right feasibility-values for the OARs, this method can easily help to reduce the dose in the OARs.

A limitation of this study was that only a dataset of single centre with a single template was explored. Further improvements of template based automatic treatment planning can be done by a planning study with multiple centres.

Other methods like knowledge based planning [3], [4] and deep learning [7], [8] are also methods that can help to obtain improvements in plan quality. The disadvantage of these methods are the need of a large training dataset to learn the model. This makes these tools less flexible for new technique or dose schemes.

This study demonstrated the feasibility of two independent dose prediction methods to personalize the automated treatment planning strategy for prostate cancer. For the personalized prostate plans a reduction of mean dose to the rectal wall was found. The use of personalized templates has the potential to improve the quality and efficiency of treatment planning for radiotherapy.

## Declaration of Competing Interest

The authors declare the following financial interests/personal relationships which may be considered as potential competing interests: The authors Martijn Kusters, Peter van Kollenburg and René Monshouwer have a Research Agreement with Philips oncology solutions. The Authors Liza Bouwmans and Karl Bzdusek work for Philips Radiation oncology systems.
