# Supplementary material for: Evaluation of two independent dose prediction methods to personalize the automated radiotherapy planning process for prostate cancer
Source: Phys Imaging Radiat Oncol. 2022 Feb 3;21:24–9. doi: 10.1016/j.phro.2022.01.006 (PMC8819373; doi:10.1016/j.phro.2022.01.006)
Supplement: Supplementary data 1 [file mmc1.docx]

**Supplementary materials**

##### Dosimetric comparison criteria

The plan quality of the PTV was assessed by the HI and the CI.

The CI was calculated by the following formula:

$$CI=\frac{V_{95\%}}{V^{PTV}}$$

whereby $V^{PTV}$ is the volume of the PTV and $V_{95\%}$ is volume of the 95% isodose surface.

The optimal value of the CI is 1. The higher the CI, the lower the conformity of the PTV in a treatment plan.

The HI was calculated by the formula of the International Commission on Radiation Units and Measurements (ICRU) [17]:

$$HI=\frac{D_{2\%}^{PTV}-D_{98\%}^{PTV}}{D_{50\%}^{PTV}}$$

$D_{2\%}^{PTV}$ = minimum dose that receives 2% of the volume of the PTV (near-maximum dose)

$D_{98\%}^{PTV}$ = minimum dose that receives 98% of the volume of the PTV (near-minimum dose)

$D_{50\%}^{PTV}$ = received dose in 50% of the PTV

The ideal value for HI is zero and it increases as homogeneity decreases.

Evaluation of plan complexity was done by comparing the number of Monitor Units (MUs). In our clinic the linear accelerators are calibrated to deliver a dose of 1 cGy/MU at the depth of dose maximum for a reference field of 10 x 10 cm^2^ to a water phantom at a source-surface distance of 100 cm.

Additional normal tissue complication propability (NTCP) calculation for the risk of rectal bleeding were estimated. The following equation was used (adapted from Schaake et al. [18]):

$$NTCP=\frac{1}{{(1+e}^{-S})}$$

Where S is defined as:

S=-8.09 + 0.23 * (anorectum(V64)) + 1.19*(anticoagulant use)

with anorectum(V64) in relative volume % and anticoagulant use is 1 (yes) or 0 (no).

Anorectum(V70) from Schaake et al. [18] has been translated into anorectum(V64) with the EQD2 formula for a 2.5 Gy fraction dose with an α/β ratio of 3 for the anorectum.

**Table S1**All plans fulfilled the clinical dose criteria shown in this table.

| ROI | Type | Primary goal dose (Gy) | Primary goal volume (%) |
| --- | --- | --- | --- |
| PTV | Min DVH (%) | 66.5 | 99 |
| PTV | Max DVH (%) | 73.5 | 5 |
| Rectal wall | Max DVH (%) | 60 | 30 |
| Rectal wall | Max DVH (%) | 30 | 80 |
| Rectal wall | Mean Dose | 45 |  |
| Bladder | Max DVH (%) | 60 | 30 |
| Anal wall | Mean Dose | 30 |  |
| L Femur | Max DVH (%) | 50 | 10 |
| R Femur | Max DVH (%) | 50 | 10 |
| Rectal wall + Anal wall | Max DVH (%) | 60 | 40 |
| Rectal wall + Anal wall | Max DVH (%) | 30 | 80 |
| sigmoid | Max DVH (%) | 60 | 30 |
| sigmoid | Max DVH (%) | 30 | 80 |
| sigmoid | Mean Dose | 45 |  |

**Table S2**

Plan comparison between clinical plans and plans based on FDVH and mFBP method. The differences in median dose metrics for PTV HI and CI, average mean dose, percentages for 30 Gy and 60 Gy volumes for rectal wall, anal wall and bladder and MUs are shown for clinical, mFBP and FDVH method. Positive values in differences indicate that these dose metric values were improved compared to the clinical plans. The data marked with an asterisk are significant results (p<0.05) between each personalized method versus the clinical method.

| **Differences in median/average dose metrics values** | | | | | | | | | | | |
| --- | --- | --- | --- | --- | --- | --- | --- | --- | --- | --- | --- |
|  | PTV HI* | PTV CI* | Rectal wall V30(%)* | V60(%)* | mean dose (Gy) * | Anal wall V30(%) | V60(%)* | mean dose (Gy) | Bladder  V60(%)* | mean dose (Gy) * | MUs* |
| clin - mFBP | 0.01 | -0.08 | 3.2 | 1.1 | 2.9 | -0.1 | -0.2 | 0.2 | -1.1 | -0.7 | -61 |
| clin - FDVH | 0.01 | -0.06 | 2.6 | 0.7 | 2.4 | -0.2 | -0.2 | 0.1 | -0.7 | -0.6 | -59 |
